# Recovery from exercise: vulnerable state, window of opportunity, or crystal ball?

**DOI:** 10.3389/fphys.2015.00204

**Published:** 2015-07-22

**Authors:** Meredith J. Luttrell, John R. Halliwill

**Affiliations:** Department of Human Physiology, University of OregonEugene, OR, USA

**Keywords:** exercise, recovery, athletic performance, regional blood flow, post-exercise, post-exercise hypotension

## Abstract

Why should we study the recovery from exercise as a discrete phenomenon from exercise itself? We identify three distinct (but not mutually exclusive) rationales that drive the need to investigate the physiology of recovery from exercise. (1) Some individuals are at a heightened risk of clinical outcomes in the immediate post-exercise period; thus the potential negative outcomes of this “vulnerable state” must be weighed against the numerous benefits of exercise training, and may be mitigated to reduce risk. (2) Many of the signaling mechanisms responsible for the beneficial effects of exercise training remain amplified during the exercise recovery period, and may present a “window of opportunity” that can be exploited by interventions to enhance the beneficial adaptations to exercise training, especially in clinical populations. (3) On an individual level, exercise recovery responses may provide investigators with a “crystal ball” ability to predict future clinical outcomes even in apparently healthy individuals. In short, the physiology of recovery is a multi-faceted and complex process, likely involving systems and pathways that are distinct from the physiology of exercise itself. For these reasons, it merits ongoing study.

## Introduction

Traditionally, the field of exercise physiology has been devoted to researching the physiological changes that occur during an acute bout of exercise, and the long-term adaptations to exercise training. More recently, the “physiology of recovery” has emerged as a sub-discipline focused on the time period between the end of a bout of exercise and the subsequent return to what is considered a “resting” or “recovered” state.

Precisely defining “recovery from exercise” is a challenging task due to the varied meanings of recovery. Recovery can refer to a distinct time frame. Depending on the physiological system or pathway of interest, this temporal definition of recovery may range from minutes (e.g., the return of heart rate to near-resting levels) to weeks (e.g., restoration of force-generating capacity after muscle damaging exercise). Additionally, these time frames vary with individual phenotype; for example, trained athletes and individuals with chronic diseases often display altered recovery time courses relative to healthy individuals. Recovery can also refer to specific physiological processes or states, which are distinct from exercise itself and resting physiological states. As a representation of what occurs during the transition from an exercising state to a resting state, we may ask, how do we enhance recovery in athletes? Lastly, recovery can refer to an end-point, e.g., having reached a state of recovery after a bout of exercise, or a starting-point, e.g., an athlete has recovered from prior training and is physiologically ready for additional training stress, or an injured athlete has recovered and can return to play.

This emerging research area, the physiology of recovery, encompasses multiple physiological systems, and is ripe for rigorous study by integrative physiologists with an interest in generating novel insights related to exercise and physical activity. Translating the basic science of recovery from exercise into practical applications related to human health and performance drives much of the interest in pursuing this intriguing (but often overlooked) aspect of exercise physiology. In this perspective, we identify three distinct (but not mutually exclusive) paradigms that drive the need to investigate the human physiology of recovery from exercise.

## Recovery from exercise: A vulnerable state?

There is substantial evidence that regular endurance and resistance exercise training reduces vulnerability to a number of chronic diseases and conditions (Booth et al., 2000). However, despite the countless beneficial effects of exercise on health and well-being, some individuals may be vulnerable to negative health outcomes (ranging from minor to life threatening) during recovery from exercise.

A dramatic example of this is the significant risk of sudden cardiac death in the 30 min following a bout of vigorous activity in men free from overt cardiovascular disease, as reported in the Physicians' Health Study (Albert et al., 2000). While this data appears alarming, these authors also note that the overall absolute risk of sudden cardiac death after exercise is quite low, with estimates of 1 death occurring for every 1.5 million bouts of exercise in men, and is even more rare in women, with 1 death for every 36.5 million hours (the difference between bouts of exercise in men and hours of exercise in women are reflective of the measurements reported in the respective studies) (Albert et al., 2000; Whang et al., 2006). The physiological mechanisms underpinning these adverse events are varied, but are likely due to cardiac abnormalities in structure or function in young individuals that lead to fatal arrhythmias, and to the disruption of unstable atherosclerotic plaques resulting in myocardial infarction in adults, particularly in previously sedentary individuals (Thompson et al., 2007). Recommended screening that includes information about previous episodes of exercise-related syncope and screening for cardiac abnormalities can identify individuals at high risk of exercise-related sudden cardiac death, especially in young athletes who are unlikely to have atherosclerotic cardiovascular disease (Maron et al., 1996; Bille et al., 2006).

Although it can be a predictor of sudden cardiac death, post-exercise syncope in the absence of structural or functional cardiac abnormalities is most often benign. An obvious example is that of prolonged dynamic exercise in warm weather, which generates a combination of blood volume loss/dehydration and elevated cutaneous blood flow that contribute to reduced venous return, predisposing individuals to orthostatic intolerance (heat syncope) during or after exercise (Hayes et al., 2000; González-Alonso, 2007). However, even in the absence of heat stress and hypovolemia, between 50 and 80% of otherwise healthy adults develop pre-syncopal signs and symptoms when subjected to head-up tilt following exercise, as recently reviewed (Halliwill et al., 2014). Both prolonged endurance and brief intense exercise predispose individuals to syncope or pre-syncopal symptoms by reducing orthostatic tolerance, a manifestation of an altered physiological state which is distinct from both the exercising state and the resting state (Bjurstedt et al., 1983; Halliwill, 2001; Halliwill et al., 2013). Briefly, post-exercise syncope (in individuals without underlying cardiac or vascular dysfunction, and in the absence of heat stress and hypovolemia) is multifactorial, involving centrally mediated sympatho-inhibition, local sustained release of a post-exercise vasodilator substance within the previously active skeletal muscle, loss of the muscle pump, and in some cases, hyperventilation induced cerebral vasoconstriction (Halliwill et al., 1996; VanNess et al., 1996; Carter et al., 1999; Kulics et al., 1999; MacDonald, 2002; Moynes et al., 2013). Research into this post-exercise phenomenon has provided insight into effective countermeasures against pre-syncopal symptoms (McCord et al., 2008; Lacewell et al., 2014). Wieling et al. (2015) have identified physical countermeasures, such as bending and contracting lower body muscles, that engage the skeletal muscle pump to augment venous return after exercise. External countermeasures, such as the impedance threshold device, which generate negative intrathoracic pressure to enhance venous return, also protect against pre-syncopal symptoms post-exercise (Lacewell et al., 2014). Lower limb compression garments, which have recently become popular among elite and recreational athletes, may also reduce pre-syncopal signs and symptoms after exercise (Privett et al., 2010).

Another vulnerable state is that of delayed-onset muscle soreness (DOMS), a common occurrence among individuals performing unfamiliar or strenuous exercise that can occur with either endurance or resistance exercise (Armstrong, 1984; Cheung et al., 2003). The muscle fiber and connective tissue damage caused by novel exercise results in a temporary decrement in muscle force development, in addition to the pain and muscle tenderness that is characteristic of this condition. The inflammatory process occurring over the following 48 h after the damaging exercise bout results in macrophage infiltration and edema which is implicated as critical in resolving the muscle damage associated with DOMS, but it is also responsible for the pain and discomfort associated with this condition (Armstrong, 1984; Smith, 1991). Complete recovery from DOMS may take weeks, but this phenomenon is complex and specific components of recovery may vary in duration. Whether this vulnerable state can be mitigated by interventions during recovery is a vibrant area of research, particularly among coaches and athletic training staff who are concerned about returning athletes to full capacity for training and competition. A number of recent articles have investigated the impact of common treatments used to prevent or attenuate soreness, including post-exercise cryotherapy and non-steroidal anti-inflammatory drugs (NSAIDs), on exercise recovery characteristics. The current consensus appears to be that cryotherapy has a negligible impact on alleviating discomfort, but may hinder the skeletal muscle repair and recovery process (Isabell et al., 1992; Paddon-Jones and Quigley, 1997; Sellwood et al., 2007). Likewise, NSAIDs may also hinder the repair and recovery, but can alleviate some of the discomfort (Urso, 2013). Support for alternative modalities such as massage or light exercise on DOMS-associated pain remains largely inconclusive, but they potentially exert a mild analgesic effect without hindering repair and recovery.

## Recovery from exercise: A window of opportunity?

For many physiological systems, recovery from exercise provides a window of opportunity to maximize or even exploit the altered physiology of the recovery period. Many of the responses we discuss here occur anywhere from 2 to 3 h immediately following exercise (e.g., post-exercise hypotension), but may last up to 48 h or more (e.g., altered blood lipids). Athletes have been taking advantage of the physiology of recovery to improve training and athletic performance during competition, by strategically consuming macronutrients during recovery. In the context of clinical populations, recovery from exercise can be exploited to mitigate the negative effects of some chronic diseases. In this section, we discuss just a few situations where recovery from exercise provides a window of opportunity to maximize the benefits of exercise.

Exercise training is a common intervention for many chronic diseases and conditions, both for the long-term training benefits, but also for the acute effects of a single bout of exercise. A bout of dynamic exercise transiently increases insulin sensitivity, decreases blood lipid levels, and reduces blood pressure after exercise, making exercise and the subsequent recovery period an ideal time for therapeutic intervention in individuals with these cardiovascular risk factors (Braun et al., 1995; Crouse et al., 1997, 1995; Grandjean et al., 2000; Holloszy, 2005; Halliwill et al., 2013). In fact, repeated bouts of exercise at least every other day have been suggested as a treatment for high cholesterol (Crouse et al., 1997). The post-exercise “window of opportunity” could be used to exploit these transient changes associated with exercise; for example, this may be a time when pharmacological interventions may act synergistically with enhanced insulin sensitivity and blunted blood lipid levels. Ideally, these interventions would slow or reverse the progression of chronic diseases, thus reducing the need for pharmacological interventions and improving quality of life in these individuals.

Our research group and others have documented the phenomenon of sustained post-exercise hypotension in young healthy adults, and the exaggerated post-exercise hypotensive response in individuals with hypertension (Rueckert et al., 1996; Pescatello et al., 1999; Forjaz et al., 2000; Halliwill, 2001; Halliwill et al., 2013). As with hypercholesterolemia, exercise training (and a single bout of dynamic exercise in particular) induced post-exercise hypotension is a proposed therapy to treat hypertension in some individuals (Hamer, 2006). Evidence from hypertensive animals models suggests that this effect may be at least partially mediated by altered gamma-aminobutyric acid (GABA) signaling in the rostral ventrolateral medulla (RVLM) and nucleus tractus solitarii (NTS), ultimately reducing the gain and range of baroreceptor activity post-exercise (Kajekar et al., 2002; Chen et al., 2009). Further exploiting this mechanism with additional interventions, pharmacological or otherwise, may be a viable treatment option in hypertensive individuals who are resistant to exercise training alone. Pro-angiogenic factors (including vascular endothelial growth factor-a, angiopoietin-2, matrix metalloproteinases) are transiently elevated after an acute exercise bout (Breen et al., 1996; Gustafsson and Kraus, 2001; Hoier et al., 2012), and may also be of therapeutic interest for patients with limited exercise capacity or impaired limb blood flow (e.g., peripheral artery disease, spinal cord injury, or muscular dystrophies). Hypoxic and blood flow restricted exercise have both been hypothesized to enhance the angiogenic adaptation to endurance exercise, although little is known about their effects on angiogenic factors when applied in the post-exercise time period (Esbjörnsson et al., 1993; Minchenko et al., 1994; Richardson et al., 1999; Olfert et al., 2001). The recovery period, when angiogenic factors are already increased, may be a window in which additional therapeutic interventions could prove more potent. It may be possible that reducing blood flow or oxygen delivery post-exercise can have an additive effect on angiogenic signaling induced by exercise alone, although to our knowledge, this has not been experimentally tested in humans.

For athletes concerned with optimizing training and performance, macronutrient intake during recovery may be a key component of their training regimen. The metabolic changes associated with both endurance and resistance exercise and recovery may be enhanced with appropriate nutrient timing strategies. In endurance athletes, maximizing skeletal muscle glycogen storage by ingesting carbohydrates in recovery has a significant effect on subsequent performance. This is taking advantage of the physiology of recovery related to glucose transporters, insulin sensitivity, and perhaps elevations in blood flow (Emhoff et al., 2011). For power and strength athletes, as well as endurance athletes, there is an analogous window of opportunity based on the elevated rate of protein synthesis in recovery, so that this time period is ripe for protein ingestion (Levenhagen et al., 2001; Areta et al., 2013). Optimization of macronutrient intake during recovery is a large area of research related to human performance, and may translate to clinical populations and older adults (Esmarck et al., 2001).

Recent evidence also suggests that interventions such as muscle cooling, applied during recovery from exercise, can enhance skeletal muscle expression of transcription factor PGC-1α, potentially promoting mitochondrial biogenesis beyond levels observed without this intervention (Ihsan et al., 2014). This finding provides a contrast to the use of cryotherapy for mitigating muscle soreness and inflammation, which was discussed above, and leads to the interesting possibility that common interventions may have divergent effects on muscle recovery, depending on the outcome variable of interest (i.e., acute inflammation vs. skeletal muscle mitogenesis). Obviously, more research focused on unraveling these complex and interconnected pathways is necessary, and may provide valuable insight into the unique physiology of recovery from exercise and how it can be exploited to improve athletic or clinical outcomes.

## Recovery from exercise: A crystal ball?

In clinical settings, exercise testing has clear and proven prognostic value, providing insight into future disease risk. Among apparently healthy and clinical populations, the physiology of recovery can also predict an individual's risk of an adverse health outcome. For example, heart rate recovery between 1 and 5 min after a moderate intensity bout of dynamic exercise is an independent predictor of all-cause mortality (Johnson and Goldberger, 2012). Both heart rate and blood pressure recovery can provide non-invasive clinical indicators related to autonomic function, making these simple measurements highly informative (Terziotti et al., 2001; Buchheit et al., 2007; Cahalin et al., 2013). In fact, measurements in recovery can non-invasively be used to assess future clinical risks that would otherwise not be apparent in a typical health screening (Cole et al., 2000; Shetler et al., 2001).

Beyond general screening functions, blood pressure recovery and post-exercise hypotension after a single bout of exercise are predictive of an individual's blood pressure response to chronic exercise training (Liu et al., 2012; Hecksteden et al., 2013). This simple, minimally invasive test could make effective use of resources in a clinical setting to identify individuals who are responsive to blood pressure reductions with training, and individuals who may require additional pharmacological intervention. In these cases, recovery from exercise provides researchers and clinicians clues to patient cardiovascular health. This concept aligns with current interest in identifying “responders” and “nonresponders” to exercise and exercise training (Karavirta et al., 2011; Timmons, 2011). With additional research in this area, it may soon be possible to identify individuals who may reap more health or performance benefits from one type of training (e.g., endurance vs. resistance training), or may also identify individuals for whom exercise would be contra-indicated (e.g., hypertrophic cardiomyopathy) (Keller et al., 2011). To our knowledge, there are currently no studies that have identified recovery from exercise variables as a means to identify responders vs. non-responders to specific exercise interventions, but this may be an interesting future direction for the physiology of recovery.

Can these notions be generalized beyond the realm of the cardiovascular and autonomic nervous systems? Relatively little is currently known about how other major organ systems recover from exercise, or how other recovery phenotypes could provide clues about either future health or even future athletic performance. For example, are there individual differences in recovery of skeletal muscle function and force development that could predict development or loss of muscle strength or function with age or training? Or could metabolic recovery predict adaptations in substrate utilization that may identify individuals for whom exercise training may prevent insulin resistance? These questions may appear far-fetched, but given what we now understand about individual responses to exercise and exercise training, it would not be surprising to discover that individual recovery from exercise is heterogeneous, phenotype-sensitive, and can be exploited for prediction of health or athletic performance benefits. It is also conceivable that the results of a simple recovery test which predicts an individual's mortality risk could provide sufficient motivation for some individuals to make lifestyle improvements to mitigate this risk. Given the relative ease of tracking heart rate and blood pressure, these recovery measurements could be made on an individual level to monitor exercise effectiveness or track health outcomes. Ideally, this information will inform future patient care through personalized medication and exercise prescription (i.e., precision exercise training). As research on the physiology of recovery expands, there is great potential for studies to find new “crystal ball” forecasters of future health.

## The trifecta of recovery

**Figure 1:**
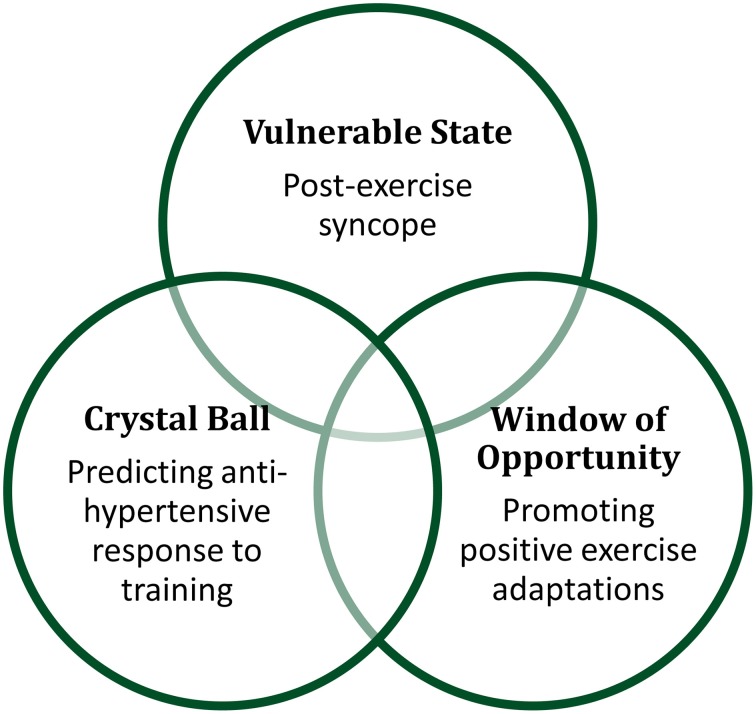
**Recovery from exercise can be conceptualized as creating vulnerable states, windows of opportunity, and providing crystal ball prognostic value**.

As evidenced by the diversity of systems engaged during recovery from exercise, this field of research has implications for both human health and athletic performance, and can be useful to researchers and healthcare professionals alike. From what is currently known about the physiology of recovery, the three paradigms we have outlined in this perspective all likely overlap within an individual after a single bout of exercise. For example, our personal interest in the sustained post-exercise vasodilation crosses all three paradigms, creating vulnerabilities and opportunities, and providing prognostic implications, as depicted in Figure 1. Such responses allow for potentially different pathways of intervention, depending on the health and goals of the individual. For example, an athlete vulnerable to post-exercise syncope may choose to perform physical counter-maneuvers to prevent syncopal symptoms, rather than pursue a pharmacological intervention that may close the window of opportunity after exercise for beneficial exercise training effects. By conceptualizing the physiology of recovery as this balance of vulnerable state, window of opportunity, and crystal ball paradigms provides a way to frame lines of inquiry and help broaden the field of exercise physiology in exciting directions for the benefit of the clinical patient, elite athlete, and weekend warrior alike.

### Conflict of interest statement

This research was funded by National Institutes of Health Grant HL115027. The authors declare that the research was conducted in the absence of any commercial or financial relationships that could be construed as a potential conflict of interest.
